# Management of a Pregnancy with a Solid Pseudopapillary Neoplasm of the Pancreas

**DOI:** 10.1155/2018/5832341

**Published:** 2018-04-16

**Authors:** Atakan Tanacan, Gokcen Orgul, Ahmet Bülent Dogrul, Fatih Aktoz, Osman Abbasoglu, M. Sinan Beksac

**Affiliations:** ^1^Division of Perinatology, Hacettepe University Medical Faculty, Ankara, Turkey; ^2^Department of General Surgery, Hacettepe University Medical Faculty, Ankara, Turkey

## Abstract

A 26-year-old primigravid patient, at 35 weeks and 2 days of gestation, was referred to Hacettepe University Hospital for pancreatic mass, giant cervical myoma, maternal systemic lupus erythematosus, thrombocytopenia, and onset of preterm labor. At 36 weeks and 1 day of gestation (6 days after admission to the hospital), regular uterine contractions started and cervical dilatation with effacement was observed. Because of breech presentation and giant cervical myoma, a cesarean section was performed on the primigravid patient under general anesthesia. Four months after the birth, subtotal pancreatectomy, partial gastrectomy, duodenectomy, cholecystectomy, and omentectomy (Whipple procedure) were performed. The pathologic diagnosis was of a solid pseudopapillary neoplasm of the pancreas; the patient was discharged from hospital after ten days.

## 1. Introduction

More than fifty percent of pancreatic cysts are composed of pancreatic cystic neoplasms (PCNs) [1, 2]. PCNs are categorized by the World Health Organization (WHO) histologic classification system into four subtypes, as follows: (1) serous cystic tumors (SCTs), (2) mucinous cystic neoplasms (MCNs), (3) intraductal papillary mucinous neoplasms (IPMNs), and (4) solid pseudopapillary neoplasms (SPNs). SPNs account for 3 percent of PCNs [3]. SPNs are symptomatic in nearly half of cases, and the most common symptoms are abdominal pain, nausea, vomiting, and weight loss [4].

SPNs reveal themselves in cross-sectional imaging modalities as mixed solid and cystic pancreatic lesions and well-demarcated, echo-poor, solid, or solid-cystic lesions in ultrasound examinations [5]. Tumor markers, like carcinoembryonic antigen (CEA), can be used for differential diagnosis, especially for MCNs. Pathological tissue specimens must be taken for definitive diagnosis [6]. SPNs are rare and arise mostly in young women [7]. SPNs have a low malignancy potential, but surgical resection is recommended because they can be locally invasive and can spontaneously rupture. In addition, distant organ and lymph node metastases can be seen in 5–10% and 2% of cases, respectively [8]. Even in the presence of malignancy complete excision and surgical debulking can provide a cure for the disease and prolong survival [7, 9, 10].

SPNs can also complicate pregnancy [8, 11–13]. However, there is limited information related to adverse pregnancy outcomes and SPNs. In this case report, we presented the management of a pregnancy that was complicated by an SPN.

## 2. Case Report

A 26-years-old primigravid patient at 35 weeks and 2 days of gestation was referred to Hacettepe University Hospital for a pancreatic mass, giant cervical myoma, maternal systemic lupus erythematosus (SLE), thrombocytopenia, and onset of preterm labor. Ultrasound examination revealed a fetus appropriate for gestational age and a 20 cm × 19 cm × 22 cm cervical myoma. The patient was diagnosed with SLE in 2014, and she was taking 200 mg/day hydroxychloroquine twice daily and 7.5 mg/day Deltacortril. The patient was in SLE remission at the time of admission to the hospital.

**Figure 1:**
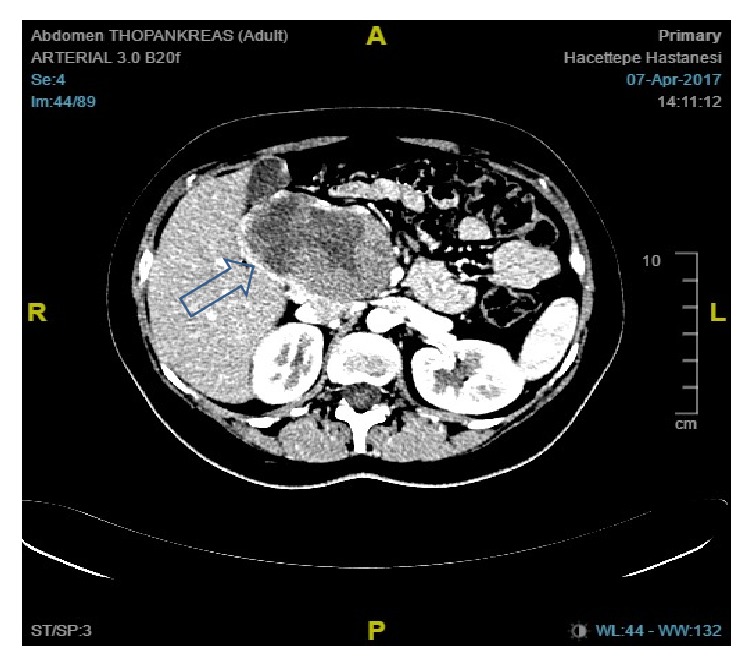
Contrast-enhanced CT of the abdomen showing the mass at the pancreatic head (arrow).

**Figure 2:**
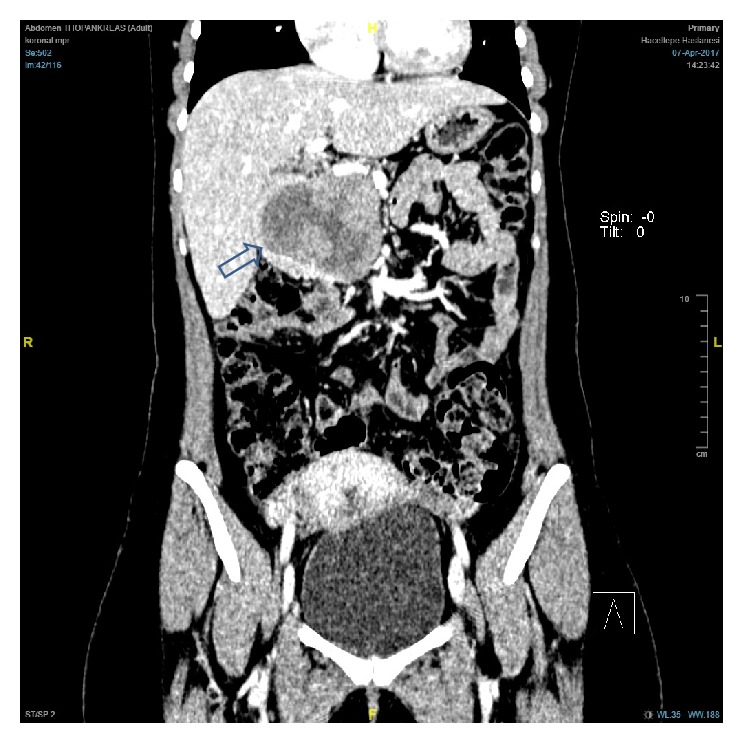
Coronal section of the pancreatic tumor (arrow).

At sixteen weeks of gestation, the patient presented at the hospital with abdominal pain, constipation, and abdominal distention. Abdominal ultrasound examination revealed a mass on the pancreas. Magnetic resonance imaging (MRI) of the pancreas was performed, and the result was reported as “9 cm × 7 cm × 6 cm neoplasm at the head of the pancreas, which might be compatible with pseudopapillary neoplasm or less probably with cyst adenoma” (Figures 1 and 2). Management of the patient was discussed in a multidisciplinary medical council which consisted of radiologists, oncologists, perinatologists, general surgeons, and neonatologists. The council decided to postpone surgery in order to stabilize the patient's SLE and to prevent iatrogenic preterm delivery. At that time, there were low amplitude contractions of the uterus, with a frequency of four contraptions every ten minutes. Routine laboratory tests found only a low platelet count of 81 × 10^3^/mm^3^. The patient consulted the rheumatology, general surgery, and cardiology departments before delivery. Four units of erythrocyte suspension (ES), 4 units of fresh frozen plasma (FFP), and 8 units of random thrombocyte (RT) suspensions were prepared. The patient was treated with intravenous hydration and bed rest in the obstetrics ward.

At 36 weeks and 1 day of gestation (6 days after her admission to the hospital), regular uterine contractions started and cervical dilatation with effacement was observed. Due to breech presentation and giant cervical myoma, cesarean section (CS) was performed under general anesthesia. Laparotomy was performed with a Pfannenstiel incision, and CS was performed using a modified anterior transverse incision above the 20 cm myoma and a 3260 g male fetus was delivered with a five-minute APGAR score of 9. A 20 cm cervical myoma was removed during the CS. Three units of ES, 4 units of FFP, and 8 units of RT were administered intraoperatively. After CS, the patient was treated at the hospital for a week and was discharged after general surgeons completed the plan for her subsequent surgery. The newborn was also discharged from the hospital without any complications.

**Figure 3:**
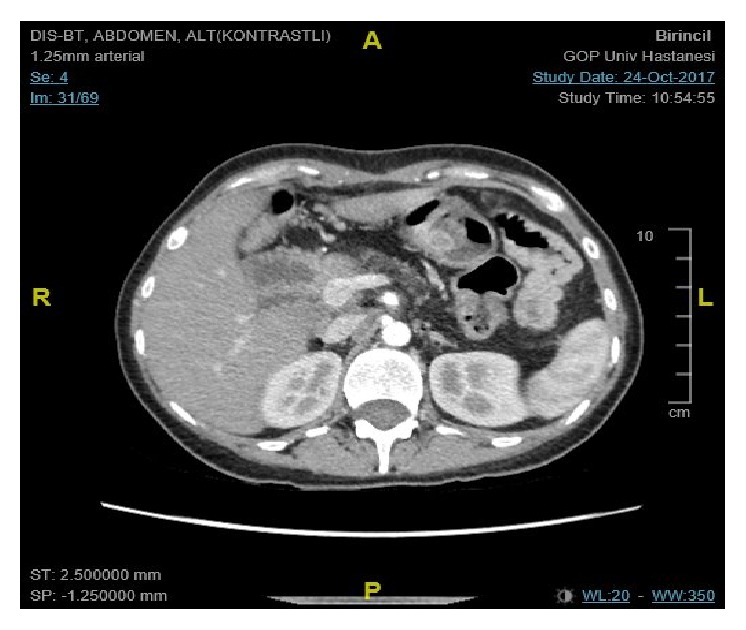
Contrast-enhanced computed tomography 7 months after Whipple procedure. No residual tumor can be seen.

Four months after the delivery, subpartial pancreatectomy, partial gastrectomy, duodenectomy, cholecystectomy, and omentectomy (Whipple procedure) were performed at the Department of General Surgery, Hacettepe University. The pathologic diagnosis was solid pseudopapillary neoplasm of the pancreas. The patient was discharged from the hospital after ten days of care. During a follow-up period of 7 months, the patient was tumor-free and in good health, without any complaints (Figure 3).

## 3. Discussion

SPNs of the pancreas are rare (2% of pancreatic tumors) and mostly affect young women in their second and third decades of life [13]. Diagnosis can be challenging, as patients present with nonspecific symptoms [13]. SPNs have low malignancy potential but can be locally invasive and spontaneously rupture. Distant organ and lymph node metastases can be seen in only 5–10% and 2% of cases, respectively [8]. Thus, surgical intervention is recommended for definitive diagnosis and prevention of complications [7, 9, 10].

Management of SPNs is more difficult during pregnancy because a balance between maternal and fetal well-being and surgical intervention for the tumor must be considered. Clinical findings, radiologic screening results, general surgery consultation, and tumor complications, along with maternal and fetal well-being, should all be assessed to determine the optimal timing of surgery. In our case, surgical intervention was postponed due to lack of sufficient evidence for the diagnosis of a malignant tumor. There is also limited clinical experience described in the literature due to the rarity of pancreatic tumors in pregnancy.

Yee et al. reported on a 39-year-old patient who was incidentally diagnosed with a large solid and cystic mass in the region of the pancreas at the eighteenth week of gestation. Noncontrast MRI identified a neoplasm at the head of the pancreas which was consistent with SPN. The patient was closely monitored for tumor growth by serial ultrasound screenings and she was delivered at term by spontaneous vaginal birth without complications. She underwent a pylorus-preserving Whipple procedure 3 months after the delivery, and the pathologic evaluation was found to be consistent with SPN. The patient was discharged from the hospital without any complication and the physicians detected no evidence of disease during her follow-up [8].

Huang et al. reported on a 29-year-old patient who was admitted to the hospital with a pancreatic mass and upper abdominal pain radiating to the back. Hypovolemic shock and peritoneal irritation signs emerged during her hospitalization. Subtotal pancreatectomy was performed for tumor bleeding and SPN was confirmed by pathology. The patient was delivered vaginally at term and no complications developed during the perinatal period. Both the mother and the infant were reported as healthy in their clinical follow-up eight months after the delivery [11].

Levy et al. reported a patient with SPN presenting with hyperemesis gravidarum. The tumor was resected surgically at the sixteenth week of gestation without complication. However, the surgical intervention did not relieve the symptoms (abdominal pain, nausea, and vomiting). Nevertheless, she was delivered near term without any complication [12].

MacDonald et al. reported on a 24-year-old patient with asymptomatic SPN diagnosed incidentally at 14 weeks of gestation. Distal pancreatectomy, splenectomy, and cholecystectomy were performed at 18 weeks of gestation. She delivered at term without complications [13].

In our case, the patient had SLE and a giant cervical myoma that complicated her pregnancy, but appropriate management by a multidisciplinary team provided optimal outcomes for both the mother and the infant. Although we have limited experience with SPNs in pregnancy, conservative management seems to be better in cases without tumor complications.
